# Immunophenotyping in routine clinical practice for predicting treatment response and adverse events in patients with MS

**DOI:** 10.3389/fneur.2024.1388941

**Published:** 2024-04-16

**Authors:** Tobias Zrzavy, Kerstin Rieder, Viktoria Wuketich, Renate Thalhammer, Helmuth Haslacher, Patrick Altmann, Barbara Kornek, Nik Krajnc, Tobias Monschein, Christiane Schmied, Karin Zebenholzer, Gudrun Zulehner, Thomas Berger, Paulus Rommer, Fritz Leutmezer, Gabriel Bsteh

**Affiliations:** ^1^Department of Neurology, Medical University of Vienna, Vienna, Austria; ^2^Comprehensive Center for Clinical Neurosciences and Mental Health, Medical University of Vienna, Vienna, Austria; ^3^Department of Laboratory Medicine, Medical University of Vienna, Vienna, Austria

**Keywords:** MS, immunophenotyping, DMD, treatment, T cells

## Abstract

**Background:**

Recent studies proposed cellular immunoprofiling as a surrogate for predicting treatment response and/or stratifying the occurrence of adverse events (AEs) in persons with multiple sclerosis (pwMS). However, applicability in real-world circumstances is not sufficiently addressed.

**Objective:**

We aimed to explore whether standard routine clinical leukocyte phenotyping before treatment initiation could help stratify patients according to treatment response or AEs in a real-world MS cohort.

**Methods:**

In this retrospective study, 150 pwMS were included, who had been newly initiated on a disease-modifying drug (DMD) and had been assessed for standard immunophenotyping before DMD initiation (baseline) and at least once during the following year. Multivariate models were used to assess an association of immune subsets and the association between immune cell profiles regarding treatment response and AEs.

**Results:**

We found that the composition of T cell subsets was associated with relapse activity, as an increased proportion of CD8^+^ lymphocytes at baseline indicated a higher likelihood of subsequent relapse (about 9% per 1% increase in CD8+ proportion of all CD3+ cells). This was particularly driven by patients receiving anti-CD20 therapy, where also EDSS worsening was associated with a higher number of CD8+ cells at baseline (3% increase per 10 cells). In the overall cohort, an increase in the proportion of NK cells was associated with a higher risk of EDSS worsening (5% per 1% increase). Occurrence of AEs was associated with a higher percentage of T cells and a lower number of percentual NKT cells at baseline.

**Conclusion:**

Immune cell profiles are associated with treatment response and the occurrence of AEs in pwMS. Hence, immunophenotyping may serve as a valuable biomarker to enable individually tailored treatment strategies in pwMS.

## Introduction

Multiple sclerosis (MS) is a chronic inflammatory demyelinating disease that affects the brain and spinal cord. Although the exact pathogenesis underlying and driving the disease is unknown, an ever-increasing number of immune-targeting disease-modifying drugs (DMDs) have been shown to reduce inflammatory-related disease activity as measured by clinical relapses and MRI activity.

However, MS is characterized by a high degree of heterogeneity both in terms of disease course and treatment response on an individual level. On the other hand, highly effective immunosuppressive DMDs carry the risk for severe adverse events (AEs) such as infections or malignancies, particularly with increasing age and treatment durations (1).

While this would suggest a personalized approach tailored to the individual patient, we currently lack biomarkers that provide adequate information for individually tailored treatment strategies, thus requiring a trial-and-error phase to find an effective and well-tolerated treatment for an individual patient.

It is well established and underlined by the success of the DMDs that perturbations associated with an abnormal immune response involving cellular interactions of peripheral immune cells trafficking into the central nervous system (CNS) are participating drivers of relapse-related mechanisms (2). Peripheral blood is an easily accessible biological sample that provides a biological “window” for assessing cellular shifts linked to DMD effects, which might offer the opportunity for predicting treatment response and/or occurrence of AEs. By identifying specific cellular markers, clinicians could develop personalized immunobiological-based treatment plans for persons with MS (pwMS), potentially leading to improved clinical outcomes.

Indeed, recent studies proposed cellular immunoprofiling as a surrogate for predicting treatment response and/or stratifying the occurrence of AEs in pwMS (3–10).

However, in contrast to protein-based biomarkers, reproducibility of these results is more difficult due to confounding influences of biological variations (such as infections, stress) or technical issues (such as sample handling and storage, cytometer setup, gating strategies, and antibody selection) (11, 12).

Here, we aimed to explore whether standard routine clinical leukocyte phenotyping before treatment initiation could help stratify patients according to treatment response or AEs in a real-world MS cohort.

## Methods

STROBE guidelines were followed in this report (13).

### Patients and definitions

For this retrospective cohort study, we used the Vienna MS Database (VMSD) of the Department of Neurology, Medical University of Vienna, which serves as both a primary and reference center mainly for Vienna and its geographical catchment area (14).

We included pwMS aged >18 years diagnosed according to the respective McDonald criteria (15, 16) who (1) had been newly initiated on a DMD between 01/01/2008 and 30/06/2019, (2) were assessed for immunophenotyping before DMD initiation (baseline) and at least once during the following year, and (3) had at least two years of clinical follow-up available.

VMSD case reports include demographic data, details of MS course (disease onset, time to diagnosis, relapses, Expanded Disability Status Scale [EDSS], and onset of secondary progression), diagnostic investigations (MRI, optical coherence tomography [OCT], cerebrospinal fluid findings) and DMT history (including initiation, interruption, changes and AEs). Data are collected retrospectively at the first visit and prospectively whenever the patient returns for scheduled (every 3–6 months) follow-up or unscheduled visits.

For the purpose of this study, the following endpoints were used for assessing treatment response: relapse (yes/no) within two years after baseline (defined as symptoms reported by the patient and confirmed by a neurologist, or signs observed by the neurologist, indicative of an acute CNS inflammatory demyelinating episode lasting at least 24 h, without fever or infection, and occurring at least 30 days after the previous relapse), EDSS worsening two years after baseline (defined as an increase by ≥1.5/1/0.5 points when the baseline score was 0/1–5.5/≥6.0, respectively).

Safety was assessed by the occurrence of AEs and severe AEs (SAEs) occurring after initiation of the respective DMD. SAEs were defined according to the Common Terminology Criteria for Adverse Events (CTCAE) V4.0 grading system grades 3 to 5, if detailed documentation was available; otherwise, they were considered as AEs. AEs were further divided into acute (infusion-related/medication intake-related) and non-acute-related AEs if appropriate.

### Immunophenotyping

For blood immunophenotyping, fresh blood was collected on EDTA-coated tubes by venipuncture and rapidly transferred to the local Department of Laboratory Medicine for analysis. Immunophenotyping was performed on FACSCanto/FACSCanto II cytometers (Becton Dickinson, Franklin Lakes, United States) by applying the following reagents (Antibodies / Clones): BD-Simultest CD3-FITC, CD16 + CD56-PE / SK7, B73.1, MY3; CD4-PerCP-cy5.5 / SK3 (Leu-3a); CD8-APC-Cy7 / SK1 (Leu-2a); CD19-PE-Cy7 / SJ25C1; CD45-V500 /2D1 (HLe-1). Lymphocytes, monocytes, and polymorphonuclear leukocytes were gated according to their forward (FSC) and side scatter (SSC) properties and based on their CD45-expression.

Following populations were gated based on the respective lineage markers: For T cells: Total T cells (CD3+), CD4 T cells (CD3 + CD4+), CD8 T cells (CD3 + CD8+), Natural Killer (NK) T cells (CD3 + CD16 + CD56+), NK cells (CD3-CD16 + CD56+) and B cells (CD19+).

For these cell types, we analyzed their absolute numbers and relative percentages at baseline as well as the relative changes in absolute numbers and percentages during follow-up compared to baseline.

### Ethics

The ethics committee of the Medical University Vienna approved the study (ethical approval number: 1968/2019).

### Data availability

De-identified data can be made available from the corresponding author upon reasonable request and after approval from the ethics review board at the Medical University of Vienna.

### Statistics

All statistical analyses and graphical representations were performed in R (Version 4.2.1). Univariate group comparisons were done by Chi-square test, Mann–Whitney U test, or independent t-test (with Welch’s correction in case of unequal standard deviations in the groups) as appropriate.

For the multivariate binary logistic regression model, the dependent variable was either relapse (yes/no), EDSS worsening (yes/no), AE (yes/no), or SAE (yes/no). Initially, all explanatory variables were tested using a univariable model, and significant variables were included in the final multivariate binary logistic regression model. Sex, disease course, and age were included in the multivariate model regardless of their significance in the univariate model due to their *a priori* potential explanatory power. The most parsimonious model was selected by using the stepwise Akaike Information Criterion (AIC) to obtain the best fit with the lowest AIC for the data set. The significance of individual variables was assessed using the Wald Chi-Squared Test.

As we anticipated a large degree of heterogeneity in our cohort due to the different DMDs used, we chose a two-step approach were we first analyzed the whole cohort regarding the endpoints and then conducted subgroup analyses for each DMD class, where a sufficient sample size was available.

We checked for linearity assumption (martingale residuals and deviance residuals) and proportional hazard assumption and found them acceptable. We tested all variables for normal distribution using the Lilliefors-test and for collinearity using variance inflation factor (VIF). Any variables with a VIF greater than 2.0 were excluded from the multivariate regression analysis. McFadden’s R squared was used to evaluate the goodness of fit for the logistic regression models. We considered a two-sided *p*-value less than 0.05 as statistically significant.

## Results

**Figure 1:**
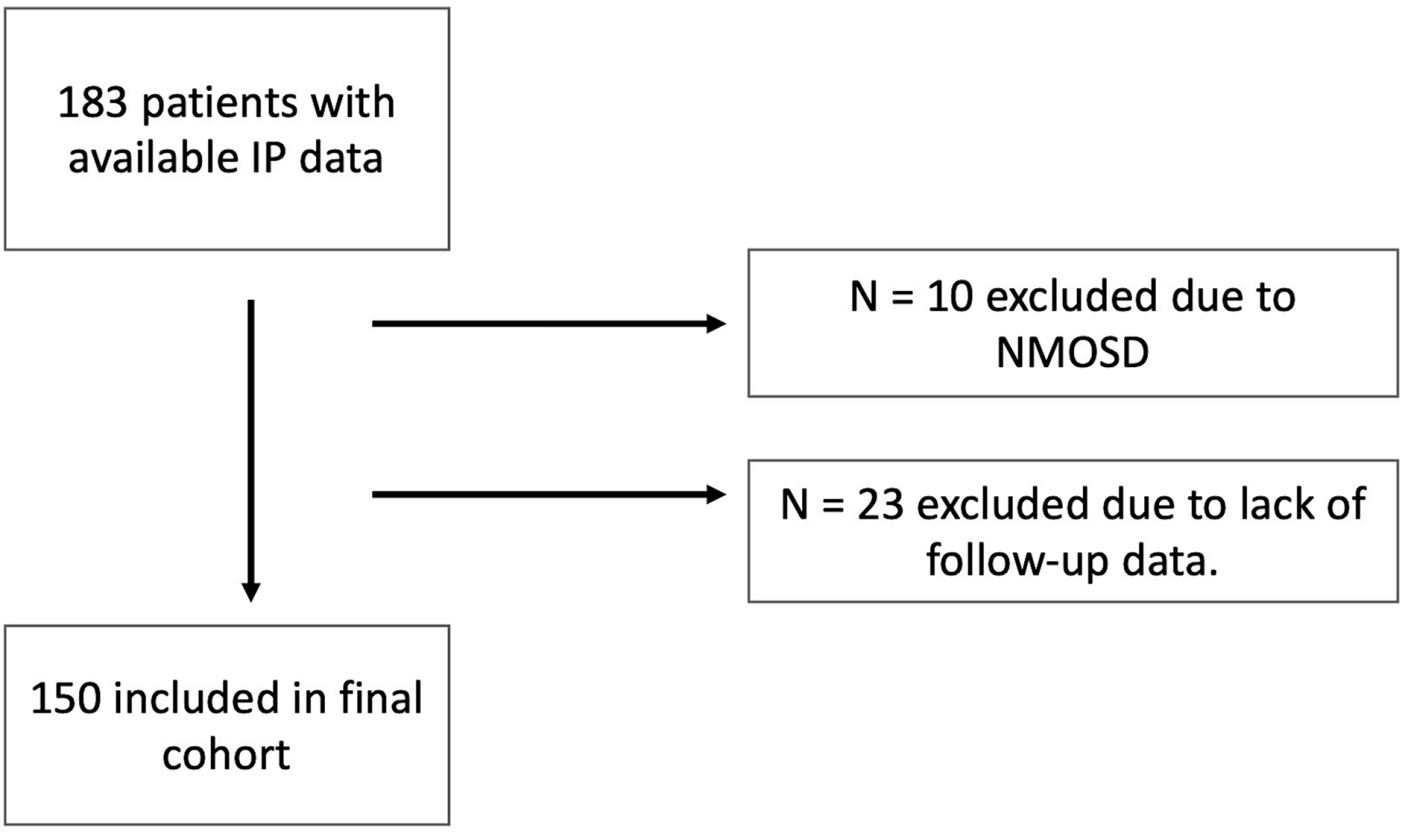
Inclusion flow chart. IP, Immunophenotyping; NMOSD, neuromyelitis optica spectrum disorders.

The detailed inclusion process is shown in Figure 1.

Demographics and characteristics of the whole cohort and the treatment groups are shown in Table 1. As expected, there were significant imbalances between treatment groups (Table 1).

**Table 1:** Characteristics of overall study cohort and DMD subgroups.

Cohort (*n* = 150)	IFN, *N* = 3^a^	GLAT, *N* = 2^a^	DMF, *N* = 25^a^	Terifluomide, *N* = 4^a^	Fingolimod, *N* = 22^a^	NTZ, *N* = 15^a^	Anti-CD52, *N* = 3^a^	CD20, *N* = 74^a^	CLAD, *N* = 2^a^	*p* ^b^
Sex										0.5
Female	3 (100%)	1 (50%)	13 (52%)	1 (25%)	14 (64%)	12 (80%)	2 (67%)	44 (59%)	1 (50%)	
Disease course										
RRMS	3 (100%)	2 (100%)	25 (100%)	3 (75%)	22 (100%)	15 (100%)	3 (100%)	33 (45%)	1 (50%)	
SPMS	0 (0%)	0 (0%)	0 (0%)	1 (25%)	0 (0%)	0 (0%)	0 (0%)	25 (34%)	1 (50%)	
PPMS	0 (0%)	0 (0%)	0 (0%)	0 (0%)	0 (0%)	0 (0%)	0 (0%)	16 (22%)	0 (0%)	
Age at Onset (y)	31 (26, 34)	26 (26, 27)	30 (26, 36)	29 (25, 32)	24 (20, 30)	24 (21, 34)	27 (21, 30)	27 (21, 39)	26 (22, 30)	0.7
Age at BL IP	31 (26, 34)	29 (28, 30)	35 (28, 42)	45 (45, 47)	38 (32, 43)	33 (30, 41)	31 (31, 32)	38 (30, 47)	38 (37, 39)	0.2
Prior DMD	0 (0%)	0 (0%)	13 (52%)	2 (50%)	20 (95%)	12 (86%)	2 (67%)	46 (62%)	1 (100%)	<0.001
Unknown	0	0	0	0	1	1	0	0	1	
Prior DMD
None	3 (100%)	2 (100%)	12 (55%)	2 (50%)	1 (5.9%)	2 (18%)	1 (50%)	27 (40%)	0 (0%)	
IFN	0 (0%)	0 (0%)	7 (32%)	0 (0%)	0 (0%)	2 (18%)	0 (0%)	2 (2.9%)	0 (0%)	
GLAT	0 (0%)	0 (0%)	1 (4.5%)	0 (0%)	3 (18%)	4 (36%)	0 (0%)	5 (7.4%)	0 (0%)	
DMF	0 (0%)	0 (0%)	0 (0%)	0 (0%)	0 (0%)	2 (18%)	0 (0%)	5 (7.4%)	0 (0%)	
Terifluomide	0 (0%)	0 (0%)	0 (0%)	2 (50%)	2 (12%)	0 (0%)	0 (0%)	0 (0%)	0 (0%)	
Fingolimod	0 (0%)	0 (0%)	0 (0%)	0 (0%)	1 (5.9%)	1 (9.1%)	1 (50%)	23 (34%)	0 (0%)	
NTZ	0 (0%)	0 (0%)	2 (9.1%)	0 (0%)	10 (59%)	0 (0%)	0 (0%)	6 (8.8%)	0 (0%)	
Anti-CD52	0 (0%)	0 (0%)	0 (0%)	0 (0%)	0 (0%)	0 (0%)	0 (0%)	0 (0%)	1 (100%)	
N.a.	0	0	3	0	5	4	1	6	1	
EDSS at BL	0.00 (0.00, 0.00)	1.50 (1.25, 1.75)	1.00 (0.00, 2.00)	2.00 (1.50, 2.88)	2.75 (1.00, 3.62)	2.00 (1.88, 3.12)	2.50 (2.25, 3.00)	3.00 (2.00, 5.00)	5.00 (4.50, 5.50)	<0.001
Time to relapse (months)	7.00 (5.00, 14.00)	11.00 (6.00, 16.00)	2.00 (0.00, 7.00)	5.00 (5.00, 5.00)	3.00 (2.00, 14.00)	14.00 (4.00, 29.00)	17.00 (17.00, 17.00)	10.00 (4.00, 15.00)	3.00 (3.00, 3.00)	0.3
Time to last FU (years)	1.75 (1.59, 5.01)	2.63 (2.31, 2.95)	4.30 (2.58, 5.13)	4.21 (3.06, 5.35)	4.53 (3.49, 6.07)	5.94 (3.50, 7.26)	3.53 (3.50, 3.79)	3.39 (2.52, 4.39)	2.52 (2.48, 2.55)	0.033
Relapse before BL IP (weeks)	1.00 (1.0, 12.00)	137.00 (72.00, 203.00)	35.00 (12.00, 73.00)	82.00 (34.00, 304.00)	47.00 (7.00, 166.00)	15.00 (9.00, 145.00)	15.00 (10.00, 109.00)	24 0.00 (12.00, 109.00)	23.00 (19.00, 28.00)	0.60
Steroids within 4 weeks before BL IP	2 (67%)	0 (0%)	2 (8.0%)	0 (0%)	3 (14%)	1 (6.7%)	1 (33%)	6 (8.1%)	0 (0%)	0.2

BL, Baseline; CLAD, Cladribine; DMF, Dimethyl fumarate; DMD, disease-modifying drugs; GLAT, Glatiramer acetate; INF, Interferon; IP, Immunophenotyping; N.a., Not available; NTZ, Natalizumab.

a *n* (%); Median (IQR).

b Fisher’s exact test; Kruskal-Wallis rank sum test.

Monocytes (*r_(p)_* = 0.32, *p* = 0.02) and NK cells (*r_(p)_* = 0.35, *p* = 0.01) correlated with age in treatment naïve patients at baseline phenotyping.

### Treatment response in the overall cohort

During follow-up, relapses and EDSS worsening occurred in 51 (34%) 44 (30%) pwMS, respectively.

Relapse occurrence was associated with a higher percentage of CD8+ T cells (OR: 1.06, 95% CI: 1.01–1.12 *p* = 0.013), which remained the only significant variable (OR 1.09, CI: 1.02–1.15, *p* = 0.021) in the multivariate model besides the progressive disease course (SPMS OR 0.31, CI: 0.10–0.99, *p* = 0.05) (Table 2). Looking only at treatment naïve pwMS, no variable remained associated with relapse activity.

**Table 2:** General efficacy.

	Relapse activity				EDSS progression			
		95% CI				95% CI		
	OR	2.5%	97.5%	*p*	OR	2.5%	97.5%	*p*
Multivariate model								
Intercept	0.16	0.01	1.83	0.14	0.18	0.03	1.125	0.08
Age	0.99	0.94	1.03	0.61	1.00	0.94	1.05	0.9
Sex(male)	0.53	0.24	1.19	0.12	0.68	0.24	1.93	0.47
Disease course								
SPMS	0.31	0.10	0.99	0.05	6.09	1.93	19.24	<0.001
PPMS	0.43	0.09	2.21	0.31	7.95	1.36	46.66	0.022
CD3 + CD8+ % of CD3 at BL	1.09	1.02	1.15	0.01				
CD3-CD16 + CD56+ % Leukocytes at 2yFU					1.05	1.01	1.09	0.012
Leukocyte count at BL
	Pseudo-*R*^2^ (McFadden) = 0.09				Pseudo-*R*^2^ (McFadden) = 0.15			

There was no association observed between temporal changes in cellular composition at one or two years after DMD initiation, and the occurrence of relapses.

EDSS worsening was not associated with any baseline immunophenotype variables. However, it was associated with a relative increase in the percentage of NK cells compared to baseline during the follow-up (per %: OR 1.05, CI: 1.01–1.09, *p* = 0.012). Expectedly, the strongest predictor of EDSS worsening was a progressive disease phenotype (SPMS OR: 6.09, CI: 1.93–19.24, *p* < 0.001, PPMS OR: 7.95, CI: 1.36–46.66, *p* = 0.022). In the cohort of treatment-naïve pwMS, only disease course was associated with EDSS progression (SPMS OR: 23.0, CI: 2.20–564.41, *p* = 0.016; PPMS OR: 9.58, CI: 1.72–65.7, *p* = 0.013).

### Treatment response in B cell depleting DMDs

The anti-CD20 group was large enough to perform a specific subgroup analysis (*n* = 74).

In this group, 17 pwMS suffered from a relapse (22.9%). In the multivariate model, the only significant variable associated with a relapse was a higher CD8+ percentage (per %: OR 1.12 CI: 1.01–1.25 *p* = 0.028) (Table 3) at baseline. Omitting progressive MS patients did not result in a multivariate model with significant associations. Excluding all pre-treated patients led to perfect separation due to the small number of cases, blocking further analyses.

**Table 3:** Anti-CD20 efficacy.

	Relapse activity				EDSS progression			
		95% CI				95% CI		
	OR	2.5%	97.5%	*p*	OR	2.5%	97.5%	*p*
Multivariate model								
Intercept	0.08	0.00	5.01	0.23	0.32	0.01	11.1	0.53
Age	0.99	0.99	0.92	1.07	0.99	0.92	1.07	0.85
Sex(male)	0.8	0.16	3.9	0.78	0.97	0.24	3.96	0.96
Disease course								
SPMS	0.4	0.08	2.06	0.27	5.81	1.15	29.51	0.033
PPMS	0.48	0.04	5.59	0.56	5.43	0.48	61.9	0.17
CD3 + CD8+ % of CD3 at BL	1.12	1.01	1.25	0.028				
CD19% of Lymphocytes at BL	0.92	0.82	1.03	0.14				
CD3 + CD8+ cell count at BL (per 10 cells)					1.03	1.00	1.06	0.039
Monocyte count at 2yFU					1.00	1.00	1.01	0.17
CD3 + CD4+ cell count at 2yFU (per 10 cells)					0.96	0.93	0.99	0.024
	Pseudo-*R*^2^ (McFadden) = 0.20				Pseudo-*R*^2^ (McFadden) = 0.29			

In the multivariate model, there was no association between temporal changes in cellular composition at one or two years after DMD initiation and the occurrence of relapses.

As in the overall cohort, EDSS worsening was associated with progressive disease course (SPMS OR: 5.81, CI: 1.15–29.51, *p* = 0.033). However, a higher absolute number of CD8+ cells at baseline (per 10 cells: OR 1.03, CI: 1.00–1.06, *p* = 0.039) as well as a lower absolute number of CD4+ T cells at the 2-year follow-up (per 10 cells: OR 0.96, CI: 0.93–0.99, *p* = 0.024) were both associated with EDSS worsening. In the cohort of treatment-naïve pwMS, only the progressive disease course remained associated with EDSS progression (SPMS: OR: 30.0, CI: 1.41–638.2, *p* = 0.032).

### Safety

AEs occurred in 78 (52%) pwMS. Most AEs were reported under anti-CD20 treatment (*n* = 31) followed by DMF (*n* = 23). Most reported AEs were acute medication intake related (*n* = 50), while the most common non-acute AEs were laboratory abnormalities and infections (*n* = 21). Of the 16 SAEs recorded, 9 were associated with acute medication intake, 4 were infections, and 3 were severe laboratory abnormalities.

Overall, AE occurrence was associated with male sex (OR: 3.31, CI: 1.179.39, *p* = 0.02), a higher percentage of T cells at baseline (CD3+) (per %: OR 1.07, CI: 1.00–1.13, *p* = 0.03), lower number of baseline percentual NKT cells (CD3 + CD16 + CD56+) (per %: OR 0.81, CI: 0.70–0.94, *p* = 0.01), as well as all but PPMS disease courses (PPMS OR: 0.05, CI: 0.00–0.55, p = 0.01) (Table 4). When looking only at infections, a higher percentage of baseline CD4 T cells was associated with the occurrence of infection during follow-up (per %: OR 1.14, CI: 1.05–1.25, *p* < 0.001).

**Table 4:** General AE.

	All AE				Infectious AE			
		95% CI				95% CI		
	OR	2.5%	97.5%	*p*	OR	2.5%	97.5%	*p*
Multivariate model								
Intercept	0.13	0.00	19.88	0.43	0.00	0.00	0.67	0.03
Age	0.97	0.91	1.02	0.20	0.96	0.89	1.03	0.25
Sex(male)	3.31	1.17	9.39	0.02	0.57	0.11	2.81	0.49
Disease course								
SPMS	0.86	0.29	2.56	0.78	3.02	0.66	13.79	0.15
PPMS	0.05	0.00	0.55	0.01	0.00	0.00	Inf	0.99
CD3-CD16 + CD56+ at 1y FU	1.00	0.99	1.00	0.08				
CD3% of Lymphocytes at BL	1.07	1.00	1.13	0.03				
CD3 + 16 + CD56+ % of T cell at BL	0.81	0.7	0.94	0.01				
ΔCD3 + CD8+ (BL vs. 1FU)	0.99	0.99	1.00	0.08				
CD3 + CD4+ % of CD3 at BL					1.14	1.05	1.25	< 0.001
Monocyte count at 2yFU					1.00	0.99	1.00	0.10
	Pseudo-*R*^2^ (McFadden) = 0.25				Pseudo-*R*^2^ (McFadden) = 0.28			

There were no significant associations with the occurrence of SAEs in the multivariate model.

### Safety in B cell depleting DMDs

In the anti-CD20 group, 12 SAEs recorded, 8 were associated with acute medication intake and 4 with infections.

The occurrence of AEs was associated with a higher percentage of baseline T cells (CD3+) (per %: OR 1.12, CI: 1.03–1.20, *p* = 0.004) and PPMS course (PPMS OR: 0.03, CI: 0.00–0.44, *p* = 0.011) (Table 5). In the multivariate model, there was no association between temporal changes in cellular composition at one or two years after DMD initiation and the occurrence of AEs.

**Table 5:** Treatment specific AE.

	AE anti-CD20				AE DMF			
		95% CI				95% CI		
	OR	2.5%	97.5%	*p*	OR	2.5%	97.5%	*p*
Multivariate model								
Intercept	0.0	0.00	0.31	0.02	9.6e10^6	3.69	2.5e10^12	0,03
Age	0.98	0.92	1.04	0.51	0.92	0.75	1.12	0.39
Sex(male)	3.24	0.88	11.86	0.08	0.91	0.07	12.5	0.94
Disease course								
SPMS	0.60	0.15	2.39	0.47	–			
PPMS	0.03	0.00	0.44	0.01	–			
CD3% of Lymphocytes at BL	1.12	1.03	1.20	0.004				
Leukocyte count at BL (per 10 cells)					0.98	0.97	0.99	0.036
CD3 + CD16 + CD56+ count at BL					0.97	0.94	1.00	0.07
	Pseudo-*R*^2^ (McFadden) = 0.26				Pseudo-*R*^2^ (McFadden) = 0.45			

Considering only infusion-related reactions, a higher percentage of baseline T cells (CD3+) (per %: OR 1.09, CI: 1.17–2.24, *p* = 0.025) was associated with a higher likelihood of suffering infusion-related reactions.

None of the variables was significantly associated with a higher likelihood of suffering from infections.

A higher percentage of baseline T cells (CD3+) (per %: OR 1.17, CI: 1.04–1.31, *p* = 0.001) was significantly associated with a higher likelihood of suffering from a SAE.

### Safety in DMF

In the DMF group, no SAEs was observed in the DMF cohort.

The occurrence of AEs was associated with lower baseline leukocyte count (per 10 cells: OR 0.98, CI: 0.97–0.99, *p* = 0.036) (Table 5). In the multivariate model, there was no association between temporal changes in cellular composition at one or two years after DMD initiation and the occurrence of AEs.

## Discussion

Individually tailored treatment strategies are needed to maximize clinical benefit as well as minimize AEs in pwMS receiving DMDs. Hence, we explored the value of standardized basic immunophenotyping in pwMS as potential cellular biomarkers for treatment response and occurrence of AEs.

We found that T cell subset composition is associated with relapse activity during treatment as a relatively increased proportion of CD8^+^ lymphocytes at baseline indicated a higher likelihood of subsequent relapse (about 9% per 1% increase in CD8+ proportion of all CD3+ cells). This was particularly driven by patients receiving anti-CD20 therapy, where the relapse risk was increased by 12% for every 1% higher CD8+ proportion of T cells. Also, a higher absolute number of CD8+ cells at baseline (3% increase per 10 cells) as well as a lower number of CD4+ cells during follow-up (4% decrease per 10 cells) were associated with an increased risk for EDSS worsening. Interestingly, an increase in the proportion of NK cells was also associated with a higher risk of EDSS worsening (5% per 1% increase).

Concerning safety, we found that a higher percentage of baseline CD4+ T cells was associated with the occurrence of infection (14% for every 1% higher CD3 + CD4+ proportion), and a lower percentage of NKT cells (19% for every 1% lower CD3 + CD16 + CD56+ proportion) was associated with the occurrence of any AEs in the overall cohort, which was confirmed in the anti-CD20 cohort. While we did not observe any SAEs in the DMF cohort, a lower baseline leukocyte count was associated with higher likelihood of any AE occurrence (2% per 10 cells).

It is well established that DMDs change the cellular immune profile in pwMS (17). Prior studies have implicated a predictive value of pre-treatment or on-treatment cellular subsets in predicting relapses and/or AEs (3, 7, 18, 19). While most studies use peripheral blood mononuclear cells (PBMCs) due to their ability to be frozen and stored, studies in fresh blood in the standardized clinical certified laboratory are rare (20).

Recently, two studies explored the potential of immunophenotyping and relapse activity in patients prior to anti-CD20 treatment. Shinoda and colleagues indicated in an in-depth immunophenotyping study that CD20^dim^CD8^+^ T cell inversely correlated with relapse activity in patients receiving ocrelizumab (3). Interestingly, Garcia and colleagues showed that CD8^+^CCR5+ T cells were inversely correlated with time since last relapse, whereas they found no correlation between CD20^dim^CD8^+^ T cells and time since last relapse (21). While this at the first sight seems contradictory to our finding of an association between a higher proportion of CD8 T cells and subsequent relapses in patients starting an anti-CD20 treatment, both groups provide evidence that these subpopulations have migratory capacity and therefore might have migrated to the CNS. Thus, predictive value of certain cell types might depend on the time of sampling in relation to application of an anti-CD20 treatment. As both studies were performed on cells collected during a rigorously controlled clinical trial, our data from a routine setting further underlines the involvement of CD8 T cells in relapse-related immunobiology specifically in patients receiving anti-CD20 treatment, although the exact sub-phenotypes and kinetics remain so far unknown.

Besides CD8^+^ T cells, we also observed an association between a relative increase in the percentage of NK cells from pre-to post-treatment and disability progression. Alterations in the proportion of NK cell subsets in MS have been previously demonstrated, but the detailed relationship is yet unresolved (22). Prior data implicated an increase in the proportions of CD56^dim^ NK cells in patients with disease progression supporting our findings (23). While we did not distinguish between NK-CD56^bright^ and NK-CD56^dim^ cells, NK-CD56^dim^ cells are the most abundant in the circulation, likely reflecting our findings. In contrast, a lower proportion NK-CD56^bright^ cell count was associated with signs of disease activity on MRI, but not there was no association with disability progression (24). Of note, these findings were reported to be independent of the treatment effect (24). However, the respective study cohort did not contain any patients receiving depleting DMDs, wherefore comparability to our cohort mostly including patients on anti-CD20 treatment is limited.

Concerning safety and AEs, a basic variant of immunophenotyping is already well-established in specific indications of clinical practice, namely the monitoring of lymphocyte counts to avoid persistent lymphopenia, e.g., in patients treated with DMF (25). However, there is a relative paucity of studies applying more advanced immunophenotyping for predicting safety and AEs in pwMS. An important concept in this regard is immunosenescence, a term referring to age-related changes in the immune system, increasing susceptibility to diseases such as malignancies and infections (26, 27). Hallmarks of immune system aging include a transition toward a memory phenotype within the T-cell compartment, which can result in a reduced ability to respond to new antigens. Additionally, there is a general decline in immune function and an increased predisposition to a proinflammatory state (28). The available evidence indicates that this phenomenon may be expedited in pwMS, leading to a higher proportion of memory CD4 T cells and impaired regulation via immune-checkpoint mechanisms (29, 30).

In line with this concept, we found that a higher percentage of CD4 T cells at baseline was associated with a higher likelihood of infections during follow-up.

Further studies with more detailed sub-phenotyping will be necessary to unravel the complex mechanisms that underlie immune aging in MS.

### Limitations

The retrospective analysis of data collected in clinical routine creates a variety of possible biases: Most important is the inherent bias of patients undergoing leukocyte subtyping depending on the choice of treatment, which is not random but determined by the characteristics and preferences of patients and the prescribing practices of clinicians. Also, different frequencies of clinical visits and different periods of follow-up might induce detection bias. As data were collected retrospectively, likely causing reporting bias, we have considered SAEs as such only if there is clear documentation and otherwise classified them as AEs. While this might have resulted in the underrepresentation of SAEs, AE rates are consistent with the published data. However, safety profiles are, in general, not comprehensively captured in retrospective studies. Further, we did not have sufficiently dense MRI data available for this study, which reduces sensitivity to detect differences in effectiveness. Immunophenotyping was based only on a very limited number of lineage markers without in-depth characterization of cellular subpopulations, each of those likely to possess subpopulations with both deleterious and beneficial properties on their own.

Additionally, we could not ascertain the time interval between the cessation of the last DMT and baseline immunophenotyping. This information is crucial as it may significantly impact baseline immunophenotyping due to potential carryover effects and long-term influences on peripheral blood phenotypes.

Our study was designed as exploratory and hypothesis-generating and, therefore, requires validation in an independent cohort.

## Data availability statement

The raw data supporting the conclusions of this article will be made available by the authors, without undue reservation.

## Ethics statement

The studies involving humans were approved by Ethics committee of the Medical University Vienna (ethical approval number: 1968/2019). The studies were conducted in accordance with the local legislation and institutional requirements. The ethics committee/institutional review board waived the requirement of written informed consent for participation from the participants or the participants’ legal guardians/next of kin because the waiver does not adversely affect the rights and welfare of participants, and the research in its entirety involves only retrospective data analysis.

## Author contributions

TZ: Writing – review & editing, Writing – original draft, Visualization, Validation, Supervision, Software, Resources, Project administration, Methodology, Investigation, Funding acquisition, Formal analysis, Data curation, Conceptualization. KR: Writing – review & editing, Writing – original draft, Data curation. VW: Writing – review & editing, Writing – original draft, Data curation. RT: Writing – review & editing, Writing – original draft, Methodology, Data curation. HH: Writing – review & editing, Writing – original draft, Methodology, Data curation. PA: Writing – review & editing, Writing – original draft, Data curation. BK: Writing – review & editing, Writing – original draft, Methodology, Data curation. NK: Writing – review & editing, Writing – original draft, Methodology, Data curation. TM: Writing – review & editing, Writing – original draft, Methodology, Data curation. CS: Writing – review & editing, Writing – original draft, Methodology, Data curation. KZ: Writing – review & editing, Writing – original draft, Methodology, Data curation. GZ: Writing – review & editing, Writing – original draft, Methodology, Data curation. TB: Writing – review & editing, Writing – original draft, Supervision, Methodology. PR: Writing – review & editing, Writing – original draft, Methodology, Data curation. FL: Writing – review & editing, Writing – original draft, Supervision, Methodology. GB: Writing – review & editing, Writing – original draft, Supervision, Methodology, Formal analysis, Data curation.
